# CRISPR/Cas9-based programmable genome editing in chickens: concepts, applications and regulatory issues

**DOI:** 10.3389/fgeed.2025.1729535

**Published:** 2026-01-09

**Authors:** Gautham Kolluri, Adnan Naim, Shiva Kumar Kurva, Jagbir Singh Tyagi, Mohd. Matin Ansari, Simmi Tomar, Ashok Kumar Tiwari, Laxmi Chouhan

**Affiliations:** 1 Molecular Physiology Laboratory, Division of Avian Physiology and Reproduction, ICAR-Central Avian Research Institute, Izatnagar, India; 2 Division of Avian Genetics and Breeding, ICAR-Central Avian Research Institute, Izatnagar, India; 3 Department of Poultry Science, Veterinary College, Jabalpur, Madhya Pradesh, India

**Keywords:** CRISPR/cas9, chicken, genome editing, gene knockout, avian influenza, bio-pharming

## Abstract

The advent of genetics, molecular biology, and genome sequencing has rapidly accelerated the development of elite genetic lines across various species, including poultry. It is now possible to introduce intra- or inter-species single nucleotide polymorphisms into chicken lines to enhance productivity. This advancement may mark the beginning of a new agricultural revolution, dramatically reducing the time required to improve poultry lines for commercial production environments. Transgenic technologies, including lentiviral vectors and piggyBac transposition, have enabled the generation of animals expressing exogenous genes. The emergence of new genome-editing tools is transforming avian biotechnology, allowing the creation of customized organisms for specific traits. Genome editing has become the most efficient method for studying gene function. First and second generation tools, such as zinc finger nucleases and transcription activator-like effector nucleases (TALENs), are limited by complex design and off-target effects. In contrast, the third generation Clustered Regularly Interspaced Short Palindromic Repeats/CRISPR-associated protein 9 (CRISPR/Cas9), represents a significant breakthrough. It encompasses guided RNA (gRNA) and the Cas9 endonuclease which together target specific DNA sequences and induces double-strand breaks that are repaired *via* error-prone non-homologous end joining, frequently causing insertions or deletions that disrupt gene function. Targeting specificity is achieved through gRNA-DNA base pairing and recognition of a protospacer adjacent motif by Cas9. Beyond gene knockout, CRISPR/Cas9 enables functional analysis of non-coding elements such as enhancers and insulators. Delivered *via* plasmid systems, Cas9 and gRNA are transiently expressed and degrade within 48–72 h, leaving no permanent genetic footprint. Since no exogenous DNA is integrated, this approach is generally considered less contentious than traditional transgenic methods in the context of genetically modified organism regulation. CRISPR/Cas9 has diverse applications in poultry, including enhancing disease resistance to avian influenza and Marek’s disease, improving productivity traits such as growth, feed efficiency, and egg-laying, and enabling early in-ovo sexing to address ethical concerns around male chick culling. It also allows control of reproductive traits for breeding management, supports bio-pharming by producing therapeutic proteins or vaccines in eggs, and facilitates functional genomics through precise gene knockouts to study development, immunity, and metabolism.

## Introduction

**FIGURE 1:**
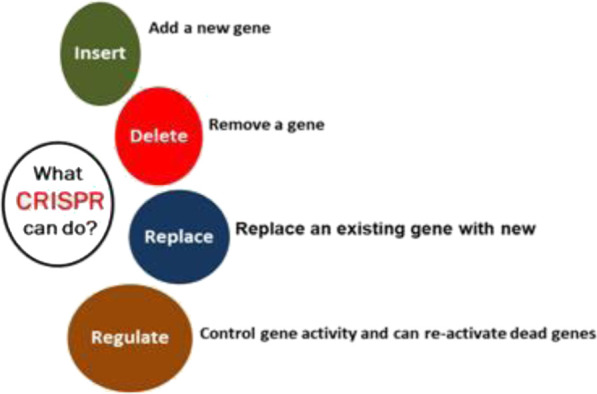
What CRISPR can do? Detailing the potential applications principles of CRISPR/Cas9 technology in animals.

The alarming growth of human population and their food and nutritional demand has created an immense pressure for quality livestock production, with improved nutritional values, more resilient to extreme climatic conditions and disease resistance (Godde et al., 2021). Poultry in particular chickens among livestock offer numerous benefits for both research and industrial applications. They are considered as the cheapest source of protein and nutrients and a mean for economic independence for the farmers. However, due to the sudden outbreaks of diseases like avian influenza virus in the past few decades has caused irreversible loss to the economy and valuable germplasm, at the national and international level. Currently, there is no existing remedy to combat such disease outbreaks as live birds remain vulnerable to viral attacks. However, genome editing in chickens and other avian has emerged as a ground-breaking tool in genetic research and agricultural innovation (Gallala, 2025). By harnessing technologies like CRISPR/Cas9, and a recent advanced version, CRISPR activation and CRISPR interference (CRISPRa/i), scientists are now able to precisely modify the DNA and regulate the expression of desirable traits such as disease resistance, and faster growth rates, which has been demonstrated *in-vitro* and *in-vivo* (Park Y. H. et al., 2020; Chapman et al., 2023). These advancements made in the CRISPR/Cas9 technology hold the potential to revolutionize poultry farming by improving productivity, animal welfare, and sustainability. Despite the promising potential, the technology also raises important ethical and regulatory considerations that must be carefully monitored (Panda and McGrew, 2021). CRISPR can be effectively implemented for studying gene function through knock-in and knock-out strategies wherein a new gene can be inserted and deleted in the genome respectively. In addition, the replacement of existing gene with new gene and regulating the gene activity through activation or in-activation principles can be ensured (Figure 1).The current review provides a comprehensive outline of principles and applications of CRISPR/Cas9 mediated genome editing in chickens.

## Programmable genome editing in chicken cells

The advent of programmable genome editing technology in biology is considered one of the most ground-breaking developments of the 21st century. Precision genome engineering (PGE) tools, which enable the rapid and precise modification of poultry genomes, have initiated a new era in the targeted breeding of poultry for food production. The ability to accurately recognize and efficiently cleave double-stranded DNA using these tools has revolutionized biotechnology. These tools not only cause gene loss-of-function but also promote homologous recombination by inducing double-strand DNA breaks (DSB). Currently, it is possible to introduce single nucleotide polymorphisms (SNPs) either within or between species into chicken lines to enhance their productivity. Inter-species SNPs refers to the difference in the single nucleotide base of a particular gene which is responsible for a characteristic trait and can be observed existing among two different species like chicken and quail; chicken and ducks, *etc.* The beneficial SNP in quail (e.g., in a growth-regulating gene), can be introduced into exact base variant (or its functional equivalent) into the chicken genome, provided that gene is sufficiently conserved and that the SNP effect is transferable. Transgenic technology, including lenti-viral systems (McGrew et al., 2004) and piggyBac transposition (Ding et al., 2005) and the second major class of recombinant DNA transposons, the Sleeping Beauty system has contributed to the development of transgenic animals for exogenous gene expression (Kong et al., 2008). Clustered regularly interspaced short palindromic repeats (CRISPR) and its associated protein 9 (Cas9) (Jinek et al., 2012) have made it easy to precisely edit genome information. Creating a DSB at the target genomic site is a critical first step in genome editing. Non-homologous end-joining (NHEJ) and homology-directed repair (HDR) are two main pathways that repair nuclease-induced DSB in almost all cell types and organisms. NHEJ can efficiently produce insertion or deletion mutations (indels) of varying lengths, which can disrupt the translational reading frame of coding sequences or affect the binding sites of trans-acting factors in promoters or enhancers. By recombining the target locus with externally supplied DNA donor templates and inducing DSBs with nucleases, HDR can be used to introduce specific point mutations or insert desired sequences. In many cases, the frequency of these alterations exceeds 1%, and in some cases, it surpasses 50%. These high rates allow for the identification of desired mutations through simple screening, without the need for drug-resistance markers.

## Cluster regulatory interspaced palindromic repeats (CRISPR): third generation genome editing

The CRISPRs and associated Cas9 proteins represent the third generation of programmable genome editing tools. CRISPR and Cas9 are prokaryotic DNA components that play crucial roles in the bacterial immune system (Lee et al., 2017a) and helps in the elimination of transduced, conjugated, or transformed foreign DNA (Barrangou and Marraffini, 2014). CRISPRs are clustered repeats found in bacteria that are identical to viral genomes (Marraffini and Sontheimer, 2008). CRISPR binds to viral RNA and disrupts it with the Cas9 protein when a virus invades a cell. Similar to ZFN and TALEN, the CRISPR/Cas9 system recognizes and induces DSB in targeted DNA sequences. The short, repetitive bases in CRISPR are separated by longer, variable sequences (called “spacers”) that have similarities with foreign DNA. Cas genes, which are preceded by an AT-rich leader sequence, frame the CRISPR array (Marraffini and Sontheimer, 2008). Cas protein, CRISPR RNA (crRNA), and trans-activating CRISPR RNA are needed for the CRISPR/Cas9 system to recognize and disrupt targeted nucleotides (tracrRNA). CRISPR/Cas9 does not require paired units to induce DSB, unlike ZFN and TALEN. Furthermore, as the synthesis of crRNA and tracrRNA is relatively straightforward, thousands of customized CRISPR/Cas9 systems for gene targeting have been developed. Due to the simplicity of CRISPR/Cas9 vector construction, this programmable genome editing tool has been applied to the vast majority of living organisms.

The most extensively investigated and developed CRISPR-Cas tools in bacteria are those of types II-A (CRISPR-Cas9) and V-A (CRISPR-Cas12a; formerly known as Cpfl) (Yao et al., 2018). The CRISPR system protects by way of three distinct mechanisms: adaptation (or spacer acquisition), expression (or CRISPR-RNA (crRNA) synthesis), and interference. During the early stages of adaptation, the foreign nucleic acid is broken up and integrated into the CRISPR locus as a spacer, primarily around the leader end, to act as a memory. Homologous to the spacer region is the sequence of foreign nucleic acid known as the protospacer. A protospacer adjacent motif (PAM) is a short CRISPR motif (1-2 nucleotides) that is located near a conserved protospacer. Cas1 and Cas2 proteins cannot select and integrate foreign nucleotides into the CRISPR locus without the presence of PAM regions. The precursor CRISPR-RNA (pre-crRNA) is synthesized through transcription in the second stage, and after maturation, it has a particular spacer sequence flanked by short RNA sequences. When trans-activating RNA (tracr RNA), RNase III, and Csnl are present, maturation occurs (Cas9). Together with trace RNA and Cas9, mature crRNA produces a complex that is essential for the ultimate stage of interference. The Cas9-crRNA-tracrRNA ribonucleoprotein (crRNP) complex forms a base pair with the protospacer, thereby activating the Cas9 protein to recognise and cleave comparable foreign nucleic acids. Since these regions are lacking in the CRISPR locus, autoimmunity is not an issue (Charpentier et al., 2015).

Essentially, CRISPR/Cas9 expression plasmids strongly express the Cas9 enzyme, but the expression vector and gRNA plasmids disappear safely after the disruption of the targeted gene due to transient expression. Thus, one of the greatest advantages of CRISPR/Cas9-mediated genetic modification is that no transgenes are integrated into the genomes of the cells or animals being modified (Lee et al., 2017c).

## CRISPR/Cas 9 and the chicken genome alteration: evidence from *in-vitro* studies

The advent of CRISPR/Cas9 genome editing technology has revolutionized the field of genetic engineering, particularly enabling precise and efficient alterations within the chicken genome. Unlike traditional methods, which were often labour-intensive and inefficient, CRISPR/Cas9 provides a programmable and robust platform for targeted mutagenesis in avian species, including chickens a model organism critical for both agricultural advancement and biomedical research. Table 1 summarises the major genes targeted in chickens to date along with their associated editing efficiencies. Véron and co-workers (2015), created the first genetically targeted chicken using a combination of CRISPR and *in-ovo* electroporation technology targeting the PAX7 gene. Similarly, C2EIP gene was targeted using CRISPR/Cas9 in DF-1 cells, chicken embryonic stem cells (ESCs) and chicken embryos (Zou et al., 2016). Chicken cell lines and embyros were used as a model for understanding their role in embryonic development and embryo related diseases (Abu-Bonsrah et al., 2016). The mammalian codon-optimized S. thermophilus Cas9 protein produced robust results in DF-1 cells, achieving editing efficiencies of up to 95% for the peroxisome proliferator-activated receptor-γ (PPAR-γ), ATP synthase epsilon subunit (ATP5E), and ovalbumin (OVA) genes when assessed using fluorescence- and puromycin-based reporters (Bai et al., 2016). The CRISPR variants Cas9-D10A nickase (Lee et al., 2017c), HMEJ-mediated site specific integration (Xie et al., 2019) and homologous recombination (Antonova et al., 2018) also yielded considerable editing success of up to 100% in somatic cells. DF-1 cells have been widely employed as a model system for studying disease resistance, bone development, and gene regulation through targeted manipulation of specific genes. They have also served as an *in vitro* proof of concept for generating relevant phenotypes. Beyond DF-1 cells, similar applications have been observed in other cell types such as primordial germ cells (PGCs) and embryonic stem cells (ESCs).

**TABLE 1:** CRISPR/Cas9 targeting different genes in chicken cells and their efficiencies.

Chicken cell types	Targeted genes	Percent efficiency	References
*In-vitro* conditions			
DT-40	*DROSHA*, *DICER*, *MBD3*, *EZH2*, *HIRA*, *TYRP1*, *STMN2*, *RET and DGCR8*	NT	Abu-Bonsrah et al. (2016)
LMH	*VNN1*	38.7	Xu et al. (2024)
DF-1 and ESCs	*Stra8*	23–25	Zhang et al. (2017)
DF-1	*PPAR- γ*, *ATP5E*, *OVA*	94.7–95.0	Bai et al. (2016)
DF-1	*C1EIS*	40	Zuo et al. (2017)
DF-1	*3′UTR of GAPDH*	89	Antonova et al. (2018)
DF-1	*TBK1*	86.67–93.33	Cheng et al. (2019)
DF-1	*tva*, *tvb*, *chNHE1*	NT	Lee et al. (2019b)
DF-1	*AMH*, *PPARG*, *TGFBR2L*	13.32–99.65	Zou et al. (2023)
DF-1	*PRMT5*	90	Zeng et al. (2024)
DF-1	*IHH*	100	Jin et al. (2025)
*In-vivo* conditions			
PGCs	*OVA*, *OVM*	≥90	Oishi et al. (2016)
PGCs	hIFN-β	22.5 & 14.5	Oishi et al. (2018)
PGCs	W83 in the chNHE1 receptor	88	Koslová et al. (2020)
PGCs	*ANP32A*	NT	Idoko-Akoh et al. (2023)

Abbreviations: DT-40, DT40 is an avian leukosis virus (ALV) induced bursal lymphoma cell line derived from a Hyline SC chicken; LMH, Leghorn Male Hepatoma; ESCs, Embryonic Stem Cells; DF-1, Chicken fibroblast cell lines; PGCs, Primordial Germ cells; DROSHA, drosha ribonuclease III; DICER, Double-stranded RNA (dsRNA) endoribonuclease; MBD3, methyl-CpG binding domain protein 3; EZH2, Enhancer of zeste homolog 2; HIRA, Histone Cell Cycle Regulator; TYRP1, Tyrosinase Related Protein 1; STMN2, Stathmin 2; RET, GF receptor tyrosine kinase; DGCR8, DiGeorge Critical Region 8; PPAR- γ, Peroxisome Proliferator-Activated Receptor-γ; TGFBR2L, TGF-beta receptor type-2 ATP5E, ATP synthase epsilon subunit; OVA, Ovalbumin; Stra8, stimulated by retinoic acid gene 8; W38, Tryptophan residue 38; C1EIS, Chromosome 1 Expression in Spermatogonial Stem Cells; UTR, Untranslated Region; GAPDH, glyceraldehyde-3-phosphate dehydrogenase; TBK1, Tank Binding Kinase-1; AMH, anti-Müllerian hormone; TGFBR2L, TGF-beta receptor type-2; VNN1, Vannin–1; PRMT5, *Protein Arginine Methyl transferases; IHH*, *Indian hedgehog; OVM*, *Ovamucin; chNHE1*, chicken Na+/H+ exchanger 1; hIFN-β, human Interferon receptor-beta; ANP32A, Acidic nuclear phosphoprotein 32 family member A NT, Not traceable.

Work done by Williams et al. (2018) reports the development of an advanced *in vivo* genome and epigenome engineering toolkit tailored for the chicken embryo model. This toolkit integrates optimized CRISPR/Cas9 components and novel vector systems to enable efficient somatic gene knockout, precise enhancer deletion, and epigenetic modulation through nuclease-deficient dCas9-effector fusions. By targeting key developmental genes the study demonstrates the toolkit’s capability to manipulate endogenous gene expression and cis-regulatory elements, facilitating dissecting gene regulatory networks during embryogenesis. Recently, Embryonic Stem Cells (ESCs) from early-stage embryos of chicken and several other avian species have been developed which were efficiently edited using CRISPR/Cas9 (Chen D. et al., 2025).

## Generation of edited chickens with specialized functionalities

**FIGURE 2:**
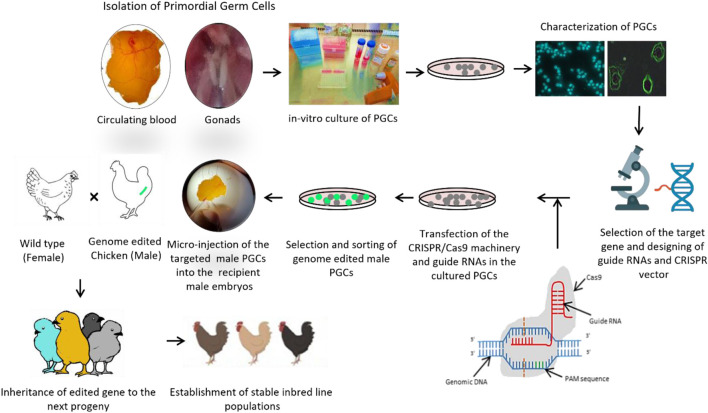
End-to-End pipeline for CRISPR editing in chickens. The pictorial representation of the whole process involved in generating a genome edited male chicken using CRISPR/Cas9 technology applicable in Primordial Germ Cells (PGCs). The pipeline briefly includes collection of PGCs from blood and gonads at 2.5 and 7 days respectively followed by further *in vitro* proliferation, propagation and characterization by employing molecular and immune-histo-chemistry, staining protocols; Designing of guide RNAs for targeted gene region, construction and validation of CRISPR vectors or RNPs; Transfection into competent PGCs followed by clonal selection and sorting of edited PGCs; Micro-injection of the edited PGCs into surrogate male embryos after destruction of native PGCs for enhanced acceptance of foreign PGCs; Hatching and rearing of recipient eggs and further screening for crossing with wild type female chicken to give rise to a progeny inheriting the edited gene. Further breeding attempts can led to the establishment of stable inbred line populations.

Mammalian systems allow for relatively straightforward embryo collection and *in-vitro* manipulation from donors, after which the modified embryos can be re-implanted into recipients (Zheng et al., 2017; Kalds et al., 2022). In contrast, CRISPR/Cas9 gene-editing strategies in birds rely on the isolation, *in vitro* culture, and propagation of PGCs (Chen X. et al., 2025), followed by their genetic modification and subsequent injection into the bloodstream of developing recipient embryos. This multi-step process substantially limits the efficiency of generating genome-edited chickens. Figure 2 depicts the detailed flow of events involved in the generation of genome edited chickens. Dimitrov et al. (2016) further advanced the field with successful germline gene editing in chickens *via* CRISPR-mediated homologous recombination in PGCs. Here, an additional loxP site was inserted into the variable region segment of the chicken immunoglobulin heavy chain (IgH) locus through homology-directed repair (HDR). Gandhi and co-workers, performed electroporation-based loss-of-function studies using CRISPR/Cas9 mechanism, in the early chick embryo to knock out Pax7 and Sox10, key transcription factors with known functions in neural crest development(Gandhi et al., 2017). Lee and co-workers (2017) studied induced loss-of-function *via* a frame-shift mutation in the CXCR4 gene in chicken PGCs as a model system. Being extragonadal, avian germ cells exhibit distinct developmental and differentiation patterns, including a unique chemokine-guided migration pathway not observed in mammals. PGCs lacking functional CXCR4 failed to migrate to the genital ridges through the circulatory route. Although the overall efficiency appeared low, the frameshift mutation caused by the disruption of two nucleotides likely contributed to a meaningful outcome in *in vivo* studies. Adenoviral CRISPR based vectors produced MSTN and melanophilin knockouts to study the loss of function in ducks and chickens (Lee et al., 2022). Knock-in strategies in PGCs for developing avian sexing model (Lee HJ. et al., 2019) and human adiponectin in egg whites (Kim et al., 2023) were also attempted. Detailed information on these functions is discussed in the subsequent sections.

## CRISPR variant, cell type and gene targeted defines the editing efficiency

CRISPR/Cas9 genome editing has become the dominant tool for functional genomics and trait engineering in poultry species. However, mutational frequencies including on-target indels, large deletions, and off-target events vary substantially across chicken cell types, delivery platforms and more importantly editing strategy. These variations arise from differences in cellular physiology, DNA repair pathway activity, chromatin accessibility, delivery efficiency, and duration of Cas9 exposure. Our critical analysis synthesizes data from recent experiments across several chicken cell and tissue types *viz.* PGCs, somatic and embryonic cell lines, and embryos, discussing both the mechanisms influencing these mutational rates and their implications for genome editing in avian models. Table 2 provides a comprehensive outlay of success of CRISPR across different cell cultures in terms of genome modification efficiency and its subsequent germline transmission.

**TABLE 2:** CRISPR/Cas9 variants: genome editing efficiency and transmission in different chicken cell culture systems.

Methods	Study model	Genome modification efficiency (%)	Germline transmission (%)	References
CRISPR/Cas9	ESCs	23–27	NA	Zuo et al. (2017)
CRISPR/Cas9	ESCs	23	NA	Zhang et al. (2017)
CRISPR/Cas9-D10A nickase	DF-1	100	NA	Lee et al. (2017b)
CRISPR/Cas9	DF-1	25	NA	Zhang et al. (2017)
CRISPR/Cas9	Chicken embryos	12	NA	Zhang et al. (2017)
CRISPR/Cas9	Chicken embryos	15	NA	Kim et al. (2023)
CRISPR/Cas9	PGCs	0–100	67–79 (48–58)	Oishi et al. (2016)
CRISPR/Cas9	PGCs	20–33	0–96 (0–48)	Dimitrov et al. (2016)
CRISPR/Cas9-D10A nickase	PGCs	35.5	27.2–56.6	Kim et al. (2020)

Abbreviations: DF-1, chicken fibroblast cell lines; ESCs, Embryonic Stem Cells; PGCs, Primordial Germ cells; Percent ranges in parentheses indicate variability between trials or separate experiments. NA, Not applicable *(in vitro)*; Value in parenthesis indicates (genome edited chickens).

A broad spectrum of mutation efficiencies ranging from 25% to nearly 100% in chicken DF-1 cells and 0%–100% in PGCs reveals the significant impact of cell type on CRISPR/Cas9 genome editing outcomes with efficiency strongly influenced by CRISPR and selection strategy, and the DNA repair mechanism at play. This wide variability underscores how the cellular context, including factors like division rate, DNA repair dynamics, chromatin state, and transfection efficiency, crucially influences both the frequency and type of mutations generated (Bai et al., 2016). DF-1 fibroblasts are particularly amenable to CRISPR/Cas9 mutagenesis, with reported mutation rates of up to 95% following selection methodologies such as puromycin enrichment, which robustly isolate successfully edited populations. The high efficiency observed in DF-1 cells has been attributed to their comparatively slower growth rate and efficient single-strand annealing (SSA) DNA repair pathway. This allows the CRISPR/Cas9 complex more time and opportunity to enact and resolve genomic breaks, leading to elevated mutation rates (Cheng et al., 2019).​ Being germ line progenitors, PGCs possess distinct cellular environment characterized by active pluripotency pathways and a unique epigenetic landscape which may either facilitate or impede successful editing events. Factors such as chromatin accessibility, cell cycle timing, and innate DNA damage response machinery play pivotal roles in determining the outcome of CRISPR interventions in these cells. In embryonic fibroblasts, the lower editing efficiency is mainly linked to poor survivability in response to transfection and antibiotic selection strategies (Kolluri, 2023).

The fidelity of CRISPR/Cas9 editing profoundly shaped by intrinsic cellular environment. For example, Cells with robust DNA repair activity particularly through pathways such as NHEJ or SSA exhibit higher frequencies of indel mutations at target loci (Ferreira da Silva et al., 2019). Higher proliferation rates and the presence of endogenous nucleases enhance the accessibility of DNA and the processing of DSB breaks, thereby boosting gene editing efficiency and indel formation (Xue and Greene, 2021). In ESCs and DF-1, gene knock-down efficiencies are in the range of 27%, comparable between these 2 cell types. The DT40 chicken B-cell line exhibits unusually high baseline homologous recombination and genome instability (Buerstedde and Takeda, 1991). Although DT40 cells often show high indel frequencies, their atypical DNA-repair landscape complicates extrapolation to *in vivo* editing outcomes. Large deletions, template switching, and microhomology-driven repair are more common in DT40 than in PGCs or DF-1 cells (Abu-Bonsrah et al., 2016). Transfection in chicken embryos, often more technically challenging, results in lower mutation rates, with efficiencies such as 15% reported for certain loci (Kim et al., 2023). Results from Lee H. J. et al. (2017) and Kim et al. (2020) demonstrated genome-editing efficiencies of 100% and 35.5% in DF-1 cells and PGCs, respectively, indicating that cell type specific factors play a critical role in governing the efficiency of CRISPR/Cas9-mediated mutagenesis. These observations underscore how experimental conditions can modulate mutation frequencies across an exceptionally broad range. Consequently, the design and optimization of genome-editing protocols must be tailored to the cellular context to achieve reliable outcomes in avian models.

Moreover, combining conventional CRISPR/Cas9 with the D10A nickase variant and homologous recombination has been shown to enhance editing efficiencies to as high as 100% (Table 2). The introduction of surrogate reporter or selectable markers (such as dual-reporter systems) can enhance the apparent frequency of targeted mutagenesis enriching for cells with successful editing up to 41.9% for nuclease-induced mutations (Wang et al., 2017). Use of chemical (e.g., Lipofectamine, Superfect or Fugene) or physical (e.g., electroporation) methods can influence mutation frequency with apparently similar efficiencies. However proper selection (e.g., puromycin) is critical for enriching edited cells and raising observed mutational frequencies.​

The literature clearly indicates that antibiotic-based selection strategies constitute a predominant factor in determining the success of genome editing events in chicken cells. Puromycin, Zeocin, Neomycin and Hygromycin are the commonly advocated antibiotics in PGC cultures for identification of cells with mutational frequencies at an effective concentration of 50 µgmL^-1^, 0.1–1.0 µgmL^-1^, 500 µgmL^-1^ and 40 µgmL^-1^ respectively. Puromycin, when used after transfection, enables strong selection for successfully edited cells, resulting in mutational frequencies approaching 100% at certain loci. Puromycin selection tends to be more stringent followed by Zeocin, effectively removing unedited cells and greatly enriching the mutated population. Neomycin selection is generally less stringent in chicken PGCs than puromycin or zeocin, occasionally allowing survival of non-transfected cells, which results in lower enrichment of mutant populations (Oishi et al., 2016).​ These antibiotics are not typically part of standard long-term PGC maintenance media but are introduced transiently following genome editing to select for desired genotypes.

Despite the intrinsic challenges of achieving efficient genome editing, the use of selection antibiotics markedly enriches for successfully edited PGCs in culture, frequently increasing observable mutation frequencies to 90%–100% when combined with optimized vector design and precise timing of selection. Importantly, PGCs exhibit cell line–specific differences in antibiotic sensitivity, and future genome-editing strategies should account for these variations to avoid cytotoxic bias and ensure reliable enrichment of edited clones. Notably, guide RNA architecture, exon choice, and gene-specific regulatory constraints exert substantial influence on editing outcomes, emphasizing the need for careful locus selection and rational gRNA design to maximize efficiency in avian systems. Selection of a target locus that includes only exonic regions—along with considerations such as exon size, the intron–exon–intron structure, and whether to use single sgRNA transfection or co-transfection of multiple sgRNAs—strongly influences genome-editing efficiency. Screening methods also play a critical role. Among these, the T7E1-based genome-cleavage detection assay is one of the most commonly used, as it provides rapid, first-line evidence of genome editing. However, detecting off-target effects at frequencies below 1% and achieving meaningful fold enrichment remain challenging (Kim et al., 2009; Kim et al., 2011). Therefore, sequencing is required as the final confirmatory analysis, ultimately improving the accuracy and reliability of measured editing efficiencies. The efficiencies are also reported to vary across founder (G0) and G1 populations.

## Applications of CRISPR/Cas 9 mediated genome editing in poultry

Genome editing technologies have revolutionized the study of genetics, enabling researchers to dissect and manipulate genomes with unprecedented precision. By deliberately inactivating or modifying specific genes, scientists can observe the resulting phenotypic changes, thereby elucidating the roles of individual genes in complex biological processes. Additionally, genome editing paves the way for therapeutic interventions in genetic disorders by allowing the precise replacement, correction, or removal of disease-causing mutations. In the field of agriculture, genome editing is transforming traditional breeding practices. Marketed as “precision breeding,” these techniques are used to introduce or enhance desirable traits, such as accelerated growth rate, increased resistance to pathogens, and improved nutritional content in crops and livestock (Tizard et al., 2016). For instance, genome editing has led to the development of disease-resistant chicken lines, improved egg quality, and poultry with enhanced growth characteristics. Furthermore, genome engineering allows producing bio-functional proteins, turning animals into “bioreactors” for pharmaceutical and industrial bio molecules.

The rapid evolution of genome editing technologies, including CRISPR/Cas9, TALENs, and zinc-finger nucleases, is also catalyzing advancements in avian biotechnology (Park J. S. et al., 2020). However, due to unique aspects of avian reproductive biology and genome organization, there is a growing demand for optimized editing tools tailored specifically to birds. Applying these cutting-edge technologies to avian species opens up a wide array of opportunities, from studying developmental biology and disease resistance to conserving endangered species and enhancing food security (Cooper et al., 2018).

**FIGURE 3:**
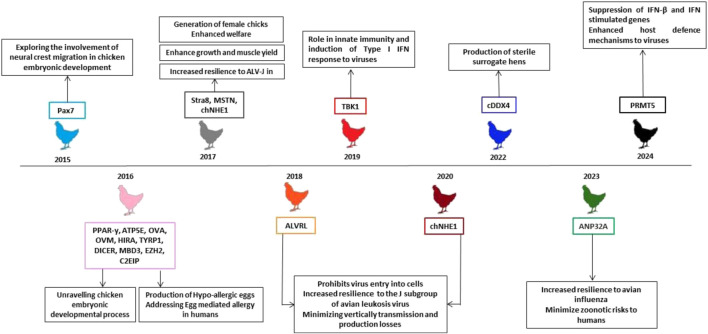
Illustration of the major milestones achieved in CRISPR/Cas9 mediated targeting of different genes using chicken cells/chicken as a model and their human significance. Since the advent of CRISPR/Cas9 gene editing technology, genes like Pax7 (2015) role was explored in neural crest migration in chicken embryo development; PPAR-γ, ATP5E, OVA, OVM, HIRA, TYRP1, DICER, MBD3, EZH2, C2EIP (2016) role was studied in chicken embryo development and also hypo-allergic eggs were produced addressing the egg-mediated allergy issues in humans. In 2017 and 2018, genes involved in Avian Leukosis Virus-J infection were targeted and also MSTN gene was targeted which is responsible for enhancing growth and muscle yield. In 2019, TBK1 gene was targeted to study its role in innate immunity and induction of type I Interferon response to viruses. In 2020, chNHE1 gene was targeted to study its potential role in developing resistance against the J subgroup of avian leukosis virus, hence containing vertical transmission and production losses. In 2022, sterile surrogate female chickens were developed by targeting cDDX4 gene which can be used as a surrogate for germ line chimera studies. In 2023, ANP32A gene was targeted which reported to enhance the resilience against the avian influenza viruses. In 2024, PRMT5 gene was targeted using CRISPR/Cas9 to study its role in suppressing the IFN-beta and IFN stimulated genes against the viral infection. Abbreviations: Pax-7, Paired Box-7; PPAR-γ, Peroxisome Proliferator-Activated Receptor-γ; ATP5E, ATP synthase epsilon subunit; OVA, Ovalbumin; OVM, Ovamucin; HIRA, Histone Cell Cycle Regulator; TYRP1, Tyrosinase Related Protein 1; DICER, Double-stranded RNA (dsRNA) endoribonuclease; MBD3, methyl-CpG binding domain protein 3; EZH2, Enhancer of zeste homolog 2; C2EIP, Chromosome 2 Expression in PGCs; MSTN, Myostatin; ANP32A, Acidic Nuclear Phosphoprotein 32A; DDX4, Dead-Box Helicase 4; chNHE1, chicken Na+/H+ exchange type 1; ALVRL, Avian Leukosis Virus; PRMT5, Protein arginine methyl transferase 5; TBK-1, Tank Binding Kinase-1; IFN-β, Interferon-beta.

Programmable genome editing thus holds immense promise in creating organisms with customized traits for agriculture, medicine, and environmental purposes. As these tools become more sophisticated, their adoption across various disciplines is expected to expand, enabling the precise engineering of genomes for targeted applications. Figure 3 shows the milestones achieved in the area of CRISPR/Cas9 mediated technology using chicken as a model organism.

### CRISPR-mediated manipulation of somatic cells

CRISPR/Cas9 nucleases have been extensively used in chicken somatic and embryonic cells. Using an enrichment system, effective gene disruptions of *PPAR*, *ATP5E*, and *OVA* were obtained in chicken somatic cells DF-1 (Abu-Bonsrah et al., 2016) and embryonic stem cells (Zhang et al., 2017). *In vivo* disruption of the *PAX7* gene in somites of early amniotic embryos was also reported (Ve’ron et al., 2015) by direct electroporation. These studies indicated varying knockdown efficiencies of 15%–41% which could be mainly relates to transfection approaches and cell systems (Zuo et al., 2017). Compared to human and mouse cells, the efficiency of customised gene editing in poultry and livestock is relatively low, which is the most significant barrier to developing transgenic animals for agriculture.

The electroporation technique, which is widely used in the chicken embryo, also results in the mosaic expression of constructs, which, when combined with loss-of-function approaches, could provide advantages compared to those observed in the fruit fly. However, gene inactivation in the chicken has been limited to knockdown by RNA interference and morpholino-based methodologies (Serralbo et al., 2013) each of which has its limitations, such as variable knockdown levels, off-target effects, and transient inhibition of transcripts.

This technology will permit the refinement of the spatial and temporal roles of genes during embryogenesis. Furthermore, this paves the way for future genetic manipulations of avian species. Indeed, recent reports of genetically modified chickens using targeted gene knockout utilising TALEN technology in isolated chicken PGCs indicate that the same strategy combined with the simpler CRISPR technique may soon be used on a large scale to generate specific genome-edited avian lines.

### Spawning only female offspring

The domestication of poultry began over 8,000 years ago, and genetics has since improved the efficiency of meat and egg producing chickens (Eriksson et al., 2008). However, because male layers grow inefficiently for meat, newly hatched males are culled soon after hatching, creating serious ethical and economic issues. The industry therefore seeks methods to identify male embryos before hatching, ideally at the time of lay. In fact, United Egg Producers pledged to end male chick culling by 2020. Sex determination in birds is well studied (Graves, 2009), but while mechanisms are understood, the exact male-determining trigger remains unclear. Gene editing now offers promising solutions by inserting marker genes into the Z chromosome. Since males (ZZ) carry two Z chromosomes and females (ZW) only one, engineered females carrying a marked Z chromosome would produce male offspring with detectable markers, while females remain unmodified (Doran et al., 2017).Recently, engineered the “Holy Grail” (HG) system to selectively block male embryo development by inserting a genetic construct into the Z chromosome. The construct includes three genes expressed sequentially: a GFP “safe-lock,” an optogenetic switch, and Noggin. Flippase excises GFP, blue light activates Cre recombinase, and Noggin expression inhibits the BMP pathway, halting male development. Two transgenic lines were generated *via* CRISPR/Cas9: one carrying HG and another expressing Flp. Crosses produced ZFlpO/ZHG-SL males whose blue light treated eggs yielded only healthy female chicks. The system ensures 100% male elimination, aligning seamlessly with hatchery practices (Cohen et al., 2023). Marker genes, such as green fluorescent protein, could allow rapid, non-invasive detection of male embryos at the lay stage, even through the eggshell using lasers similar to candling. This enables efficient sexing before incubation. Other selectable genes could also be applied, combined with “null segregant” approaches already standard in plant breeding (Venkatesh et al., 2016).This strategy eliminates the wasteful “hatch-and-cull” cycle, producing only wild-type laying hens for eggs while allowing improved handling, incubation, and nutrient recovery from males. The result is improved production efficiency, higher food quality, and more ethical farming practices.

### Increasing the growth rate of chicken

Myostatin (MSTN), a member of the TGF-superfamily, serves as a well-known negative regulator of myogenesis during skeletal muscle development by inhibiting the proliferation and differentiation of myoblasts. It is predominantly found in muscle tissues and plays a crucial role in regulating both embryonic development and the maintenance of adult tissue homeostasis (Doran et al., 2017). The absence of MSTN leads to excessive skeletal muscle growth, highlighting a significant mechanism that controls muscle size in healthy individuals. In mice lacking the myostatin gene, muscle fiber hyperplasia and hypertrophy resulted in a dramatic increase in skeletal muscle mass, producing animals approximately twice the size of their wild-type counterparts (Lee and McPherron, 2001). Successful CRISPR/Cas9-mediated gene editing to knock out the myostatin gene has been reported in sheep (Han et al., 2014) and medakafish (Yeh et al., 2017). Successful editing of the chicken MSTN gene was achieved both *in vitro* and *in vivo* using the D10A nickase variant of CRISPR/Cas9, resulting in the absence of MSTN protein expression *in vitro* (Lee J. H. et al., 2017) and, *in vivo*, chickens exhibiting increased breast and leg muscle mass along with reduced abdominal fat deposition (Kim et al., 2020). Interestingly, targeted MSTN mutagenesis resulted no detectable change in either cellular morphology (Lee H. J. et al., 2017) or body weight in knockout chickens in comparison to wild-type counterparts (Kim et al., 2020).

### Uncovering the causes of egg allergies

Egg allergy is the most common food allergy in children, affecting up to 2% of the population and being the second most common IgE-mediated food allergy in infants and young children after cow’s milk allergy (Sicherer and Sampson, 2006; Rona et al., 2007). It is estimated that between 0.5% and 2.5% of young children develop egg-specific IgE antibodies in response to egg allergens (Rona et al., 2007). A population study conducted in Australia revealed that 8.9% of infants are allergic to chicken eggs (Osborne et al., 2011). Osterballeand co-workersreported a prevalence rate of 1.7% for food allergies on their list (Osterballe et al., 2009). Ovomucoid (Gal d1), ovalbumin (Gal d2), ovotransferrin (Gal d3), lysozyme (Gal d4), and albumen (Gal d5) are the major allergenic proteins in chicken eggs, with Gal d1–d4 found in the albumen and Gal d5 in the yolk (Tan and Joshi, 2014). Additionally, the egg yolk proteins apovitellenin I and IV cause allergic reactions (Tey and Heine, 2009). Egg allergy corresponds to food allergy type I, which is distinguished by atopic dermatitis, eosinophilic esophagitis, and cell-mediated disorders. Although ovalbumin constitutes approximately 54% of egg allergens and ovomucoid is the predominant allergen (Caubet and Wang, 2011). Due to its high heat stability, resistance to proteolytic enzymes during digestion, and potent allergenicity, OVM is more difficult to eliminate than OVA (Bernhisel-Broadbent et al., 1994). Therefore, gene disruption of egg white allergen genes such as OVA and OVM has the potential to reduce allergenicity in eggs, thereby reducing immune responses in individuals who are allergic to egg white–containing foods and vaccines. By inducing PGC mutations with TALEN, Park et al. (2014) created chickens with a specific gene knockout aimed at the OVA gene. This study describes the successful generation of heterozygous OVA gene mutant offspring in which nucleotide deletion mutations of ORF shifts led to the loss of OVA gene function. This was the first study to demonstrate the efficacy of site-specific nuclease-mediated genome-editing technology in producing mutant chickens. Oishi et al. efficiently (>90%) mutagenized OVA and OVM cultured chicken PGCs using plasmids encoding Cas9, a single guide RNA, and a gene encoding drug resistance, followed by transient antibiotic selection. In their experiments, neomycin-treated cells led to more efficient mutations of these two genes (34% in OVA and 13% in OVM). By transplanting mutant-ovomucoid PGCs into recipient chicken embryos, homozygous and heterozygous mutants with OVM gene disruptions were generated. Eggs produced by hens with homozygous mutations in these allergen-encoding genes may be tolerable for those with egg white allergies. It is possible, albeit time-consuming, to cross OVA and OVM mutant chickens to produce lines with mutations in multiple allergy-related genes. Alternatively, given the efficiency of CRISPR/Cas9 mutagenesis in chicken PGCs, it may be possible to generate hens with the disruption of multiple allergenic genes more quickly by disrupting them simultaneously in PGCs. This method of manipulating the chicken genome to produce allergen-free eggs is an exciting new way to combat egg allergies and provide a healthier, safer egg-eating experience for those who are allergic to them.

### Modifications in egg biochemical composition

Few concepts have been developed to demonstrate the potential role of CRISPR/cas9 in the production of “designer eggs”, or eggs whose biochemical composition has been modified to meet a specific need. Egg lipid content could be modified by targeting several key regulators in the form of very low-density lipoproteins (VLDL) in the ovary (Schneider, 2009). It has been reported that a chicken strain carrying a single mutation at the VLDLR locus, the “restricted ovulator,” or R/O, lays small eggs very infrequently, accounting for only about 2% of the eggs laid by wild-type hens (Elkin et al., 2006). As a result of their inability to effectively deposit VLDL and VTG into their oocytes, mutant females develop severe hyperlipidemia and atherosclerosis-like characteristics. Roosters that are VLDLR-/VLDLR+ (carriers) have a normal lipid metabolism. VLDLR females, which represent a model for an oocyte-specific receptor defect leading to familial hypercholesterolemia, are sterile due to their inability to produce eggs. Eggs with low cholesterol were produced by the increased expression of genes encoding for cholesterol 7 alpha-hydroxylase (Cyp7a1) and apolipoprotein H (ApH) during the synthesis of bile acid and cholesterol efflux. While the decreased levels of very low-density lipoprotein receptor (VLDLR), apolipoprotein B (ApoB), apovitellenin 1 (ApoVLDlII), and vitellogenin (VTGI, VTGII, and VTGIII) genes expressed in the ovary are indicative of atherosclerosis, they do not explain the disease (Zhou et al., 2014). Given its role in regulating cholesterol levels, Pcsk9 is a useful proof-of-concept, and this gene was successfully knocked out with CRISPR/Cas9, with the effects lasting for 6 months (Science Daily, 2018). Oishi et al. (2016) recently successfully (>90%) mutagenized two major egg allergens, namely ovalbumin (Gal d2) and ovomucoid (Gal d1), in cultured chicken primordial germ cells (PGCs) *via* transfection using plasmids encoding Cas9, a single guide RNA, and a gene encoding drug resistance, followed by transient antibiotic selection. In their experiments, neomycin-treated cells caused a more efficient mutation of these two genes (34% in *OVA* and 13% in *OVM*). By transplanting CRISPR-induced mutant-ovomucoid PGCs into recipient chicken embryos, homozygous and heterozygous *OVM* gene disruptions were generated. Chickens with homozygous mutations in these allergen genes produced eggs with low allergenicity, which a person with egg white allergies could tolerate.

### Chickens with built-in disease resistance

Poultry disease outbreaks are a serious threat to the commercial poultry sector and can cause catastrophic losses for both developed and developing nations’ economies. Pandemic infections like avian influenza (AI) and avian leukosis virus (ALV) have had a significant negative impact on the poultry business and the general public. The avian influenza virus (AIV), which may quickly reassort and become hypervirulent, that can cause unforeseen pandemics with high mortality rates (Suarez and Schultz-Cherry, 2000). Depending on the age, health, and antigenic distance of the virus, the effectiveness of existing vaccination techniques, which employ live or inactivated viral vaccine strains to suppress AIV in poultry, is either restricted or non-existent (Barber et al., 2010). Therefore, the development of long-term, effective strategies to control new diseases is important and has long been a priority.

Another objective of avian CRISPR/Cas9-based genome editing technology is disease-resistant bird lines. Breeding for disease resistance presents a big challenge for the chicken industry. Although genetic variation is one of the main factors determining resistance, the practical uses in poultry have not yet been identified. We may now analyse genetic, epigenetic, and transcriptome differences within a species or closely related species to find factors linked to disease susceptibility and resistance because of the quick growth of genomic resources and next-generation sequencing. For instance, the Fayoumi chicken is renowned for being immune to several diseases. According to recent studies, the gene expression of two different genetic lines of the same species, the Leghorn and Fayoumi chickens, reacts differently to AIV infection (An et al., 2019). Breeding disease-resistant chickens may be made easier with more investigation into these differently expressed genes and the use of gene editing technology to introduce those notable genomic changes into the chicken genome. Since prior studies have indicated that overexpression of short hairpin RNA, which precisely regulates viral RNA polymerase expression, may limit AI transmission, genome editing is one of the most promising approaches for disease resistance in avian species.

The W38 site of the chNHE1 receptor is the primary binding site for ALV-J infection, and its disruption can generate resistant chicken lines. Precise, targeted disruption of tva, tvb, and chNHE1 receptor genes has been reported to enhance resistance to ALV subtypes A, B, and J by interfering with viral entry through frameshift-induced loss of receptor function. Notably, the proportion of infected DF-1 cell clones was significantly reduced in all knockout lines except tvb (Lee HJ. et al., 2019), indicating that frameshift mutations in tvb did not confer the expected level of resistance. This suggests that a more precise HDR-based editing strategy may be required to achieve full resistance. LentiCRISPR-mediated generation of W38 chNHE1 knockout PGCs resulted in markedly reduced p27 antigen titers and increased env expression following ALV-J infection compared with wild-type controls (Zhou et al., 2025). *In vivo* studies further demonstrated that chickens carrying this knockout exhibited enhanced resistance to ALV-J, as reflected by lower viraemia (proviral copy numbers) and reduced p27 titers (Koslová et al., 2020; Kheimar et al., 2021). Similarly, homozygous frame-shifting indels in the tva, tvc, and tvj receptors introduced premature stop codons that conferred resistance to ALV subtypes A, C, and J, respectively, in DF-1 cells (Koslová et al., 2018).

A notable example that helps illustrate disease mechanisms is the discovery of the chicken ANP32A (chANP32A) protein as a species-specific host factor required for the polymerase activity of AIV and defining a barrier to zoonotic transmission (Park Y. H. et al., 2020). They found that chicken ANP32A alone and not ANP32B and ANP32E, plays a key role in facilitating vPol activity of these viruses. Additionally, they also found that the human ANP32C, ANP32D, and ANP32E have suppressive effects on vPol activity as compared to human ANP32A and ANP32B. Building on this, Idoko-Akoh et al. (2023) applies genome editing to explicitly block AIV replication and transmission in chickens by targeting critical ANP32 family members. Their results demonstrate that while edited birds exhibit strong resistance to infection, complete protection is only achieved by ablating the entire ANP32 family, revealing the redundancy among isoforms exploited by the virus for escape and adaptation. Unlike human ANP32A (huANP32A), the chicken version contains an additional 33 amino acid segment, which significantly enhances the viral polymerase activity in avian cells (Long et al., 2019). Using gene editing technologies to replace the chANP32A gene with the huANP32A gene may lessen the AIV increased polymerase activity in chicken cells, giving the birds illness resistance.

A further intriguing route for learning and maybe identifying gene editing targets for disease resistance is the comparative genomics of chickens and ducks. Despite being related to chickens, ducks have quite distinct innate immune systems and levels of tolerance to AIV infection (Smith et al., 2009; Blyth et al., 2016). Due to their lack of the RIG-I gene, chickens have been demonstrated to be more vulnerable to AIV infection than ducks (Barber et al., 2010). It is possible to create hens with increased influenza resistance using genome editing by precisely introducing RIG-I or RIG-I-like “natural” disease-resistance genes into chicken genomic safe harbour regions. Furthermore, HIV-related mutations in the CCR5 host receptor gene have offered genetic evidence in support of this strategy. Simple *in vitro* and *in vivo* testing of altered creatures would be made possible by genome editing of these receptors; this research may aid in the creation of disease-resistant chickens. Recently, the role of T cells in chicken immunity has been elucidated through the development of T cell-knockout chickens (Heyl et al., 2023). Together, these studies significantly advance understanding and practical applications for controlling avian influenza: the mechanistic clarity informs rational gene editing strategies, and experimental genome editing demonstrates the potential and complex requirements for generating truly resistant chicken lines.

### Enhancing climate resilience

Climate change, particularly global warming, is a significant factor adversely affecting poultry livestock production and product quality. According to St-Pierre and colleagues, the poultry industry suffers an estimated annual economic loss of USD 126–165 million due to heat stress and other climate-related challenges (St-Pierre et al., 2003). Heat stress alone significantly reduces egg production and deteriorates meat quality (Wasti et al., 2020). Traditional breeding methods have struggled to keep pace with these rapidly changing environmental pressures. There is an urgent need to develop location specific climate resilient varieties which could be possible through investigation of the transcriptomic profiles of climate resilient species *viz.*, indigenous chicken breeds and other poultry species such as ducks, quails, guinea fowl, and turkeys to explore the novel genes mediating stress mechanisms. These species are considered valuable for their innate tolerance and survival under adverse climatic conditions and disease outbreaks. Exploring the potential for silencing, up-regulating or introducing genes associated with thermo-tolerance using CRISPR/Cas9 technology offers a promising solution. These genes can potentially be introduced into more vulnerable breeds to enhance their resilience. Recent work by Rachman et al. (2024) on the genomic analysis of Nigerian indigenous chickens identified key thermo-tolerance and immune response genes, including *cytochrome* P450 2B4-like, TSHR, HSF1, CDC37, SFTPB, HIF3A, SLC44A2, and ILF3. Heat shock proteins (HSP70, HSP90) and thyroid hormone receptors have already been recognized as central to the thermal physiology of chickens. With the advent of CRISPR/Cas9, the expression of these heat-responsive genes can be precisely modulated, thereby enhancing the capacity of birds to maintain cellular function under heat stress.

### Egg-expressed biopharmaceuticals and the role of CRISPR/Cas9 in avian bioreactors

The market value of biopharmaceuticals has been steadily increasing due to the quick advancements in biotechnology. Avian bioreactors have been explored to produce specific recombinant proteins in eggs (Zhu et al., 2005) *via* embryonic microinjection of retroviral or lentiviral vectors. However, these early approaches often failed to achieve heritable phenotypic gene expression in eggs, likely due to factors such as transgene silencing, ectopic expression, or insertional mutagenesis disrupting host genes. For pharmaceutical proteins to be produced inexpensively, more than 300 eggs must be laid by hens each year. There are roughly 10 major protein types found in chicken eggs, which makes it easier to separate and purify target proteins. Furthermore, medicinal compounds made from chicken eggs may be more bioactive because of the similarities and differences between the protein modifications in chicken, such as the N-glycosylation pattern, and those in humans. According to reports, transgenic chicken can generate therapeutic proteins such ß-galactosidase and ß-lactamase, interferon-a-2b, interferon-ß-1a, granulocyte-colony stimulating factor, human FSH, human erythropoietin (EPO), and human EGF. This is based on the previously described advantages (Penno et al., 2010; Park et al., 2015; Shi et al., 2020; Mukae et al., 2021; Xie et al., 2024).

In the recent times, CRISPR/Cas9 genome editing system combined with germline-competent PGCs, allowing the targeted integration and expression of exogenous genes in ovarian cells. These genes are expressed in egg whites, typically under the control of the OVA promoter, yielding 0.5–3.4 mg of protein per egg (Penno et al., 2010). Using a knock-in (KI) strategy, cDNA constructs are precisely introduced into PGCs *via* the CRISPR/Cas9 system to produce germline chimeric roosters. These roosters can transmit the transgene, leading to the generation of transgenic hens that lay eggs containing the protein of interest (Park et al., 2015). Notable achievements include the production of monoclonal antibodies (mAbs) in the egg white, with yields ranging from 1.4 to 1.9 mg/mL of albumen. These mAbs are among the most effective biopharmaceuticals used in treating a wide range of human diseases, including cancer, autoimmune disorders, inflammatory conditions, and infectious diseases (Mukae et al., 2021).

Recent advancements include the production of human adiponectin (ADPN), a hormone derived from adipose tissue with therapeutic potential for insulin resistance. Gene-edited chickens have been engineered to express ADPN at yields ranging from 0.59 mg to 4.59 mg per egg (Kim et al., 2023; Yoo et al., 2024). Additionally, human interferon-beta (hIFN-β), known for its antiviral and antiproliferative effects, has been produced in egg whites at concentrations of 1.9–4.4 mg (Oishi et al., 2018). Proteins used in laboratory research have also been successfully expressed in eggs of CRISPR-edited chickens, with yields between 4.96 and 9.86 mg. Enhanced Green Fluorescent Protein (EGFP) and Green Fluorescent Protein (GFP), commonly used as visual markers to validate genetic modifications, have also been produced (Harel-Markowitz et al., 2009). Altogether, the findings suggest chicken bioreactor as an efficient alternative, scalable and reliable recombinant protein production system (bioreactors) (Meng et al., 2025; Mozdziak and Petitte, 2004; Lillico et al., 2007). By combining targeted genome editing with stable germline transmission, gene edited chickens offer a promising, cost-efficient system for generating high yields of therapeutic and laboratory-grade proteins in egg whites.

## CRISPR activation (CRISPRa) and CRISPR interference (CRISPRi)

The CRISPR-Cas9 system has revolutionized biological research by enabling highly precise manipulation of gene expression. While initial focus was on genome editing—inducing targeted DNA breaks—newer techniques have emerged that can modulate gene activity without altering the DNA sequence itself (Pickar-Oliver and Gersbach, 2019). The most notable of these are CRISPR activation (CRISPRa) and CRISPR interference (CRISPRi), which allow researchers to turn genes on or off in a controlled and reversible manner (Chen and Qi, 2017; Heidersbach et al., 2023; Gallala, 2025). Both CRISPRa and CRISPRi use a variant of the Cas9 enzyme, called catalytically dead Cas9 (dCas9). Guided by specific RNAs (gRNAs), dCas9 binds to target DNA sequences but does not cut them. Instead, dCas9 serves as a platform for recruiting regulatory domains: CRISPRa: dCas9 is fused with transcriptional activation domains (such as VP64, VPR, or p300), stimulating gene expression. CRISPRi: dCas9 is fused with repressor domains (such as KRAB or KRAB-MeCP2), suppressing gene expression. This system allows programmable and sequence-specific modulation of gene activity, making it a powerful tool for research and biotechnology (Chapman et al., 2023; Han et al., 2023).

Recent studies have demonstrated efficient use of CRISPRa and CRISPRi in chicken DF-1 fibroblast cells. Researchers tested various constructs to either activate or repress gene expression where dCas9 linked to VP64, VPR, or p300 has been used to increase expression of target genes. Similarly, to study repression, dCas9 alone or fused with KRAB or KRAB-MeCP2 domains has been deployed to silence genes (Chapman et al., 2023).The position of gRNAs relative to the transcription start site (TSS) significantly affects the efficiency of both activation and repression. For instance, CRISPRa has been used to identify and validate transcriptional enhancers, aiding in mapping regulatory elements in the avian genome. Moreover, genes important for immune response (e.g., *HMGA1*, *SMARCB1*, *IRF7*, *PPARG*) have been studied using these tools to probe antiviral defence without permanent genome changes (Chapman et al., 2023).Additional systems, such as SAM (Synergistic Activation Mediator) for activation and LSD1 (lysine-specific histone demethylase 1) domains for repression, have been applied in chicken embryo studies. The VPR activator domain successfully enhanced expression of *OVA*, a major egg white protein, in embryonic fibroblasts and DF-1 cells. dCas9-VP64 was also used for activating viral genes in chicken lymphoblastoid cell lines, showcasing the broad utility of these approaches in poultry gene regulation (Chapman et al., 2023).

The expanding CRISPR toolkit offers the potential to develop animals with improved productivity, disease resistance, and tailored egg composition, alongside addressing animal welfare concerns. In chickens, CRISPR/Cas9 systems have already contributed to advances in these areas. However, there is a need for more comprehensive evaluation of different effector domains and gRNA placements within the CRISPRa/i toolkit to fully harness these technologies for avian research and practical applications (Han et al., 2023; Gallala, 2025).

## Ethical issues and regulatory approvals for CRISPR/Cas9

The advancement of genome editing in farm animals is accompanied by a range of complex ethical, and regulatory challenges. Ethical and regulatory considerations include questions about animal welfare, the intrinsic value of animal life, and the potential consequences of altering genomes in ways that may not align with traditional farming practices or public sentiment. Acceptability concerns further complicate the scenario, as consumers, advocacy groups, and policymakers may harbour reservations about the safety of products derived from genome-edited animals, as well as their broader environmental and health impacts (Singh and Ali, 2021).

Addressing these multifaceted issues is essential for the responsible and effective progression of genome editing technologies. Overcoming ethical, and regulatory, challenges will be pivotal in enabling the development of farm animals with enhanced productivity traits, such as higher yields, improved disease resistance, and greater adaptability to climate change. The regulatory environment for gene-editing technologies like CRISPR/Cas9 is complex and varies widely between regions. Many countries are still developing comprehensive frameworks to address the unique challenges these technologies present. Issues such as the difficulty of tracing genetic modifications and the blurring of lines between natural and genetically altered organisms require a re-evaluation of existing regulatory strategies (Cormick and Mercer, 2019).

Globally, nations have adopted two primary frameworks for regulating genome-edited organisms, including farm animals such as chickens: the process-based and product-based regulatory systems. The approach taken in each country significantly influences the ease with which genome-edited animals can be developed, approved, and marketed (Cormick and Mercer, 2019; Bartkowski et al., 2018; Fernández Ríos et al., 2025).

### Process-based regulatory system

In a process-based regulatory system, oversight is determined primarily by the techniques used to create the genetic modification, regardless of the final properties or traits of the edited organism. Under this approach, if genome editing technologies such as CRISPR, TALENs, or ZFNs are used—even if no foreign DNA remains in the resulting organism—the product is subject to strict genetically modified organism (GMO) regulations.

European Union: The European Court of Justice ruled in 2018 that organisms modified by mutagenesis techniques, including recent genome editing methods, fall within the scope of GMO legislation (Court of Justice of the European Union (CJEU) (2018)). As a result, any animal or plant produced using genome editing technologies is subjected to the same stringent approval, labeling, and traceability requirements as traditional GMOs, regardless of the final product’s genetic composition.

New Zealand: New Zealand maintains a highly precautionary, process-based system. The Environmental Protection Authority (EPA) classifies all organisms created or altered through modern gene-editing technologies as GMOs, demanding comprehensive regulatory approval.

### Product-based regulatory system

Conversely, the product-based regulatory system focuses on the attributes of the final organism rather than the technologies used in its production. Regulatory scrutiny applies mainly to whether the new organism contains foreign genetic material (transgenes) or exhibits novel traits that might pose safety or ethical risks.The U.S. Food and Drug Administration (FDA) and U.S. Department of Agriculture (USDA) have adopted a product-based approach for genome-edited animals and plants. The agencies evaluate genome-edited products based on their end characteristics. If the genome-edited animal is free of transgenes—meaning it does not contain DNA from a different species, and the changes could be achieved through conventional breeding—it is generally not classified as a GMO and may be subject to less stringent regulation. However, if foreign DNA remains in the organism, the product falls under traditional GMO oversight.

Many countries in South America (e.g., Argentina, Brazil), and parts of Asia have followed the U.S. lead, adopting regulations that emphasize the final product’s attributes over the specific editing methods used. Recently, Argentinian company has produced the world’s first genome edited foals using CRISPR mediated genome editing technology. Argentina’s National Advisory Commission on Agricultural Biotechnology (CONABIA) has evaluated proposed gene edited animals that do not contain any foreign DNA or a new combination of genetic material and judged them to be exempt from GM regulation. These include gene edited applications in fish (tilapia), cattle, and horses (Fernández Ríos et al., 2025; Wray-Cahen et al., 2024). However, The International Federation of Horseracing Authorities (IFHA) and the International Federation for Equestrian Sports (FEI)3 have prohibited gene doping.

As of now, there have been no reported commercialized LM animals in South Korea; only experimental research has been announced. The current regulatory framework in the country defines terms related to genetic modification, modern biotechnology, and GMOs using criteria set in bio-safety guidelines. However, the existing regulations limit the definition to recombinant DNA, excluding comprehensive coverage of the latest new biotechnology advancements. Specifically, genetically modified animals are defined as those whose genetic material has been altered by recombinant DNA technology, encompassing modified gametes, embryos, and limited to cultured cells derived from fetal or adult organs. This implies that animals modified using advanced new biotechnology techniques like gene editing might fall outside the scope of genetically modified animals (Van Eenennaam et al., 2019; Lim and Choi, 2023; Wray-Cahen et al., 2024).

In India, as per the National Guidelines for Stem Cell Research (2017) of the Indian Council of Medical Research (ICMR), Department of Health Research (DHR) and Department of Biotechnology (DBT), Genome modification including gene editing (for example by CRISPR-Cas9 technology) of stem cells, germ-line stem cells or gamete and human embryos is restricted only to *in-vitro* studies. It will require thorough review by the Institute Committee for Stem Cell Research (IC-SCR), Institute Ethics Committee (IEC) and Institute Bio-Safety Committee (IBSC), and finally by Review Committee on Genetic Manipulation (RCGM). So far no permission has been granted for the release of any GE animal in commercial market.

In UK, genome-edited animals for food require ethical consideration and specific regulatory permission; however, a recent change in law in England (the Genetic Technology (Precision Breeding) Act) allows for the commercial development and sale of precision-bred food. The Nuffield Council on Bioethics emphasizes the need for strong public dialogue and clear welfare standards to guide the responsible application of this technology. According to EU law, organisms whose genomes have been modified using the CRISPR/Cas method are to be considered GMOs. Their use would therefore require a genetic engineering approval and, under current law, they could only be kept in a genetic engineering facility. Free-range husbandry would then be equivalent to a release project. Without appropriate legal adjustments, mass use would certainly be inconceivable. In the UK, genome-edited animals for food require ethical consideration and specific regulatory permission; however, a recent change in law in England (the Genetic Technology (Precision Breeding) Act) allows for the commercial development and sale of precision-bred food.

### Implications

The divergence between process-based and product-based systems creates a patchwork of regulatory environments internationally. This affects global research collaborations, commercialization strategies, and consumer acceptance of genome-edited animals. For example, a genome-edited chicken line developed in the U.S. and considered non-GMO could face significant regulatory hurdles if exported to the EU. Safety and risk assessment are crucial when regulating gene-edited poultry products. The main risk is off-target effects, which could result in unintended genetic changes with unpredictable consequences (Dimitrov et al., 2016). Long-term effects on animal health and the environment must also be carefully evaluated. Some studies suggest that precise CRISPR/Cas9 editing can, for example, confer disease resistance in poultry without apparent adverse effects (Koslová et al., 2020). However, ongoing monitoring and periodic reassessment are vital to identify and mitigate risks promptly. The involvement of bio-safety and bio-security experts is equally important to address the dual-use potential of gene editing technologies.

## Conclusion

Genome editing in livestock represents a transformative leap in agricultural science, offering the potential to significantly enhance the efficiency, sustainability, and welfare of animal farming. Using cutting-edge tools like CRISPR-Cas9, scientists can now make precise genetic modifications in livestock species, including chickens, to improve traits like disease resistance, growth rates, reproductive efficiency, and meat quality. This technology not only promises to boost productivity and reduce the environmental footprint of livestock farming, but it also opens new avenues for addressing global challenges such as food security, food safety and climate change. Besides, the implications of genome edited chickens on humans are multifaceted in terms of addressing the challenges of antibiotic resistance, egg-based allergy, reducing zoonoses, production of leaner poultry meat cuts and eggs enriched with omega-3 fatty acids. While the potential benefits are vast, genome editing in chickens also raises important ethical, regulatory, and societal questions that will shape the future of animal breeding and agriculture.
